# Public Health and Unconventional Oil and Gas Extraction Including Fracking: Global Lessons from a Scottish Government Review

**DOI:** 10.3390/ijerph15040675

**Published:** 2018-04-04

**Authors:** Andrew Watterson, William Dinan

**Affiliations:** 1 Occupational and Environmental Health Research Group, Faculty of Health Sciences, University of Stirling, Stirling FK9 4LA, Scotland, UK; 2 Communications, Media & Culture, Faculty of Arts & Humanities, University of Stirling, Stirling FK9 4LA, Scotland, UK; william.dinan1@stir.ac.uk

**Keywords:** unconventional oil gas extraction, fracking, public health policy, global

## Abstract

Unconventional oil and gas extraction (UOGE) including fracking for shale gas is underway in North America on a large scale, and in Australia and some other countries. It is viewed as a major source of global energy needs by proponents. Critics consider fracking and UOGE an immediate and long-term threat to global, national, and regional public health and climate. Rarely have governments brought together relatively detailed assessments of direct and indirect public health risks associated with fracking and weighed these against potential benefits to inform a national debate on whether to pursue this energy route. The Scottish government has now done so in a wide-ranging consultation underpinned by a variety of reports on unconventional gas extraction including fracking. This paper analyses the Scottish government approach from inception to conclusion, and from procedures to outcomes. The reports commissioned by the Scottish government include a comprehensive review dedicated specifically to public health as well as reports on climate change, economic impacts, transport, geology, and decommissioning. All these reports are relevant to public health, and taken together offer a comprehensive review of existing evidence. The approach is unique globally when compared with UOGE assessments conducted in the USA, Australia, Canada, and England. The review process builds a useful evidence base although it is not without flaws. The process approach, if not the content, offers a framework that may have merits globally.

## 1. Introduction

Unconventional oil and gas extraction (UOGE) includes fracking for shale gas, coal bed methane extraction and underground coal gasification. Fracking involves drilling wells and then hydraulically fracturing (fracking) shale rock seams with large quantities of water and additives at high pressure to extract shale gas or coal bed methane. There are two competing schools of thought about UOGE. Depending on suitable geology, the extractive industry views onshore oil and gas reserves as an important resource that should be exploited, arguing that shale gas is both a significant feedstock and key part of the energy mix in the transition to a low carbon economy. Those advocating UOGE also believe industry and government are capable of effective regulation and that the technology poses little threat to public health. Those opposing UOGE view fracked shale gas as at best a very short-term energy solution. Moreover, they consider fracking may present a range of short-, middle-, and long-term risks to local communities, national and global public health. These critics often point to the availability and desirability of more sustainable energy sources and policies [1,2]. UOGE creates hazards including public health risks through, air, water, soil pollution as well as seismic activity. One independent large scale UOGE review in 2016 on UK public health implications found serious gaps in knowledge of the potential impacts of exposure, health, socio-economic effects, and climate change along with “some concerning signals in the literature and legitimate uncertainties derived from first principles” [1] (p. 1). Noise and radiation risks and threats to well-being and mental health have also been identified [2]. Debates about the public health costs and benefits of UOGE do not exist in isolation from considerations about UOGE impacts on jobs, the local economy, agriculture, house prices, insurance, and tourism. In contrast to the Scottish government position, the UK government is pressing ahead with fracking based on a review of evidence and public health impact assessment produced by Public Health England (PHE) [3] that is more limited in terms of breadth and depth than the assessment of evidence relied on by the Scottish government. The policy being pursued in England appears to lack meaningful measures from the UK government or industry to secure a social license for UOGE activities. Such an approach parallels the policies adopted in parts of North America and Australia [4]. 

**Figure 1:**
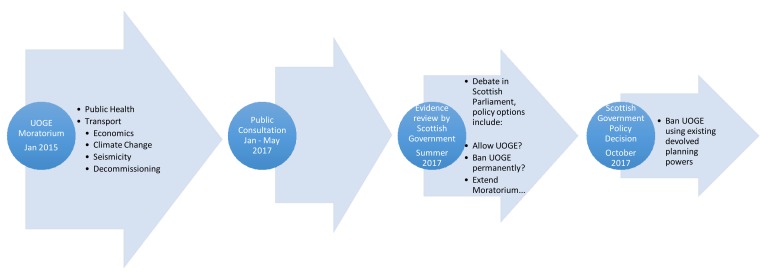
The Scottish UOGE moratorium: process and timeline.

The principal aim of this paper is to examine the evidentiary and consultative processes underpinning Scottish government UOGE policy relating to public health. The Scottish government presently controls planning and environmental pollution regulation but not workplace health and safety, which is currently still under the control of the UK government. Westminster is also currently responsible for licensing gas exploration and development. No fracking or other large scale commercial UOGE has ever occurred onshore in Scotland. The Scottish government imposed a moratorium on onshore UOGE activities in 2015 and set up six enquiries to examine evidence on the industry including a specific request to look at mitigation measures if UOGE was approved. These reports have been recently published (Table 1). The enquiries were designed to be taken together, along with a public consultation exercise, and were to be used by the Edinburgh government to decide whether to allow onshore UOGE in Scotland. In terms of breadth, depth, and scale, this approach appears more detailed than any undertaken to date globally (see Section 3 below for detail on this two-year review process). The public health impact assessment in particular is underpinned by what appears to be a rigorous and transparent examination of existing scientific literature drawing on external peer review at some stages. Given that public health and environmental impacts are often cited as the two key concerns surrounding development of UOGE, it is perhaps surprising that the Scottish government did not commission an in depth environmental impact analysis on a par with the HIA (environmental impacts were partly subsumed under the economics, climate, and seismicity strands of the review). The Scottish review of evidence on UOGE offers a coherent evidence-based and inclusive approach with citizens as well as scientists, civil servants and politicians involved (Figure 1). The public consultation exercise closed at the end of May 2017 and the Scottish government took the decision not to approve UOGE including fracking of shale in October 2017 based both on its commissioned reports and the public consultation exercise that solicited submissions from the public, researchers, and community groups. Final parliamentary approval will follow a strategic environment assessment of the government’s new policy. Integral to the success of such processes, and the stability of the associated policy position among stakeholders, is the quality, independence, rigor, and comprehensiveness of the evidence. Another signal feature of the Scottish review has been the holistic and integrative approach adopted. In practice this means the avoidance of fragmentation of the assessment decision into silos, and full engagement with the public to ensure that a social license exists for any actions taken. Additionally, research published since the six enquiries were completed in late 2016 should also be considered by policy makers (although it is not clear whether this has happened). The process used by the Scottish government, as well as the organization and results of the respective investigations, albeit most relevant and sometimes particular to Scotland, provides a useful generic framework for UOGE public health and related assessments across the globe. At a national level the Scottish case appears to be unique.

## 2. Materials and Methods 

The paper examines, from a process and public health perspective, how the Scottish government addressed:

(1) industry demands and planning applications, after the UK government granted licenses to extract UOGE in Scotland, to develop unconventional gas extraction which includes fracking but also coal bed methane and underground coal gasification;
(2) “public demands” for the health and other related consequences, including direct and indirect costs, of UOGE to be assessed;
(3) the existing evidence base on public health and related reports from other jurisdictions on UOGE either to inform national, regional or project specific “health impact assessment” decisions.

The methods used involved analysis of policy documents and technical, industry and regulatory reports including the mechanisms for the review. The study includes an examination of the policy background to the Scottish UOGE moratorium, and an analysis of all the major relevant reports and publications produced or commissioned by the Scottish government on UOGE. It draws on related relevant scientific and policy papers, especially critiques of the health impact assessments available within and beyond Scotland on UOGE, along with an assessment of the regulatory process used and briefly considers key comparisons with approaches used elsewhere in the world. A detailed account of our analysis of the peer reviewed evidence base on fracking regulation and public health, including our assessment of key policy documents relation to UOGE development in the UK and Scotland is already published [4,5]. As well as drawing on the available documentary evidence the authors have been monitoring the public and policy debate on UOGE in Scotland for several years. The analysis offered below is also informed by our participation in public meetings addressing UOGE development across Scotland, attendance at industry events and political meetings where UOGE was under discussion, private meetings and interviews with advocates involved in lobbying and campaigning around UOGE Scotland. We have also systematically monitored Scottish mass media, specialist media outlets, corresponded with officials, journalists and public health experts working on issues related to UOGE and followed key protagonists on social media. We have reviewed data on UOGE disclosed via Freedom of Information requests, and well as evidence provided to official planning tribunals. This detailed background knowledge of the UOGE issue culture in Scotland has informed the analysis, but the evidentiary bedrock of this article rests on the content of the documents and official reports cited, and our assessment of the process of deliberation. Thus, we offer some remarks on the dynamics of the Scottish policy process, to contextualize how scientific evidence and scenarios for the development of UOGE articulated with public and political will formation. 

There are some international and national frameworks for carrying out “health impact assessments”. However, there are no such agreed frameworks or indeed statutory requirements in most countries for assessing new or old technologies at a national level, for example, in the energy field and for making policy. Such policy decisions may be informed by health impact and other assessments, but these do not dictate central and local government decision-making. All parties of course tend to claim their assessments of hazards and risks are evidence-based or evidence-informed but that is not always how policy is made [6,7]. It presupposes the same evidence base is used by all parties and interpreted in the same ways. Scientists and engineers have recently put forward the view that they should help to inform public deliberations, be they local or national, on fracking risks but accept research “cannot, on its own, resolve all controversies” [8]. 

There may therefore be merit in examining reports and papers available on fracking that attempt to bring together some of the key elements used in health and other impact assessments. These may provide a valid approach to the policy-making process and have some generalizability within limits. Nearly all reviews include some analysis of fracking impacts on air and water [1,9,10]. In addition to universal recognition of the potential impacts in these areas fracking research is constantly throwing up new findings that affect risk assessments and it would be difficult for this reason to compare the evidence from some reports from 2012 with publications from 2017 [11,12,13,14,15,16]. Different countries and regions may also be faced with very different geological, economic, social, and other conditions that could impact on policy decisions. For this reason, the focus of this paper is on the processes that may inform policy decisions about fracking.

The publications listed in Table 2 have been selected using the following criteria: (1) did the documents cover those topics most relevant to informing policy on key health and health-related issues; (2) what was most relevant to the UK and Scottish context; (3) what were the most widely cited and influential policy documents used by governments across the globe (based on our reading of the literature which includes reviews at different scales including international, national, and regional [4,5]); (4) what publications from other parts of the world attempted to bring together a range of evidence to inform fracking policy decisions. We recognize that these outputs were designed for a range of “policy” purposes and prepared with different resources, timetables and terms of reference but are nevertheless of use in assessing “process”. Some of these publications consider local-, regional-, or state-based fracking proposals. Some of the publications specifically excluded various topics as not relevant to their brief. However, the publications listed all provide in our view the best available corpus to identify a more comprehensive approach to the policy-making process. 

For each publication, we examined its core contents to identify the key areas they commonly addressed and how comprehensive and detailed the coverage of those areas was. This allowed us to compile the list of headings for Table 2. It also allowed us to make an assessment at the end of the exercise of the most comprehensive, “integrated” and detailed publications that could be used to inform policy processes. The headings which form an umbrella for a public health and related topic assessment therefore appear to provide the basis for a comprehensive assessment of fracking. The rationale for their inclusion is provided next.

(a) Occupational Health. Worker health and safety should be an integral part of any public health assessment of fracking in terms of exposure to risks. It could involve large numbers depending on wells drilled. Drillers may be the canaries of the industry. Checking on occupational health and safety where exposures may be wider and higher and longer than those experienced by the rest of the population could provide a means of assessing possible longer-term impacts on local communities.
(b) Climate change. This impacts on global public health through methane emissions but also through emissions of other constituents, such as gasoline and diesel involved in extraction, production and distribution of shale gas and will directly or indirectly affect national, regional, and local health—in some places through flooding or fire. Linked to climate change and shale gas are potential public health problems: for example, shale gas use in plastics production and the impacts of plastic products. These are not currently considered in either policy or health assessments but may be in the future. A process ensuring independent and rigorous assessment of what fracking industry regulation does or can do in practice is again necessary.
(c) Regulation. How exactly fracking is conducted, monitored and how safe or hazardous it is, may partly depend on regulations, enforcement, and regulatory resources. Therefore, assessments of regulation are critical not just to mitigation but to the policy approval process.
(d) Industry practice. This has been suggested as a major factor in determining the public health threats of fracking and its acceptability, although the intrinsic technology and related materials along with the geology and hydrology of sites may sometimes be potentially greater factors. A process ensuring independent and rigorous assessment of what the fracking industry does in practice is again necessary. The role of consultants and independent experts in preparing health impact assessments for industry merits attention when assessing the policy process. In the UK, such consultants have prepared industry guidance at a national level on how fracking might be done safely.
(e) Economic factors. Economic benefits or economic damage, linked to employment and trade, from the fracking industry may have positive and negative impacts on public health. Some governments may weight these factors more highly than public health in their policy decisions on the industry. Although difficult to measure and often with a large range of impacts, a detailed assessment of the economics is justified based on independent assessments open to scrutiny.
(f) Vulnerable populations. These are of particular concern to bodies such as the WHO as well as many national governments. These populations may be disproportionately disadvantaged or theoretically benefitted by fracking. This flags the need for policy decisions to consider short-, medium-, and long-term effects of fracking—something relevant to all the headings listed so far. Hence it is important their position is addressed in the assessment process.
(g) Social determinants of health. These merit inclusion and are a public health policy priority for many governments as well as municipal bodies. They are connected to a much bigger picture of social and economic development and decline especially regarding “boomtown” effects.
(h) Peer review. Peer review should automatically occur with scientific journal papers but does not always happen with industry, commercial consultancy, or government reports. The necessity for such independent review is critical to the confidence that can be placed in findings. Several but not all the publications we examine rightly distinguished between peer reviewed and non-peer reviewed literature. Reports not only need to be perceived as open to such scrutiny but should demonstrate that they have undergone that process. This is one important mechanism in helping agencies and the public to have confidence in material used to make policy. The absence of peer review would not necessarily invalidate a publication, but its presence will usually enhance its credibility and should perhaps be integral to any policy process assessment.
(i) Declaration of interests. This is needed to guarantee transparency in the policy making process and will ensure any conflict of interests in the preparation of fracking assessments is made clear at the outset. Again, the absence of a declaration of interests when there are competing interests would damage a publication, but its inclusion would not necessarily do so and hence such declarations should be viewed as essential to the policy evaluation process.
(j) Public engagement. There is widespread recognition of the importance of consulting with various publics who may be impacted by the development of UOGE. From a governance perspective the outcomes of policy and regulatory decision making are seen to be more stable and legitimate if key stakeholders are consulted about proposals, in a timely and meaningful manner. In theory this can lead to effective partnership working and co-regulation by stakeholders. In relation to public health the inclusion of communities at early stages in the planning and during research phases of HIAs is not only considered best practice but can help identify and calibrate public health issues among various communities and vulnerable populations.

## 3. Results

### 3.1. The Context

Scottish politics has witnessed an unprecedented period of campaigning and activism since the independence referendum in 2014, which marked a notable high point in political participation in Scotland. In the wake of that referendum there have not only been a series of elections (for Westminster in 2015 and 2017, and for the Scottish Parliament in 2016) and the Brexit referendum, but also mobilization and vigorous campaigning by community and national groups on several “hot” issues, including fracking. 

Given this wider context, the Scottish government (like other governments) faced a series of challenges and tensions when taking decisions on UOGE that might have uncertain outcomes. These included economic, legal, and political as well as technical and even chronological dilemmas. The government clearly attempted to balance a series of competing interests and potential benefits and costs. Public health was a central consideration in this balancing act and weighing of evidence. 

Potential benefits from UOGE development might include possible increases in, or contributions to, economic growth and employment, greater tax revenues and direct and indirect investment across the country, including in areas of particular disadvantage. If such benefits were to materialize at scale these in turn could impact positively on public health. However, the development of fracking could potentially be a threat to air, water, soil, and roads, physical and mental health, and well-being. UOGE development might also negatively impact on other areas of economic activity, notably agriculture and tourism. Other associated costs include harming Scotland’s “green” image and undermining climate change targets. The downsides of UOGE development also come with associated adverse public health effects. Legal challenges might emerge to any decisions on UOGE. Politically, most of the Scottish parties contained both pro and anti-fracking supporters. Any government decision on UOGE was likely to antagonize either a large number of communities or powerful industry interest groups. Among those with technical expertise, opinion on the desirability of UOGE varied. There were scientists, commercial consultants, and academics as well as industry who supported UOGE and scientists, academics, and environmental groups as well as communities who either were unconvinced about the technology or opposed to it. In terms of timetables, the Scottish government in the early 2010s was being pressed to decide a course of action on delayed planning decisions.

Following the UK government decision to promote fracking and grant exploration and development licenses for fracking across the UK in 2008, the Scottish government was faced with a dilemma. This was partly related to the constitutional division of powers between the UK and Scotland, with the former responsible for licensing exploration and the Scottish government having devolved control of planning, along with local authorities across Scotland. The government in Westminster has powers relating to the auctioning and distribution of exploration licenses across the UK. However, local authorities are responsible for granting planning permission and consents for drilling wells in their areas. In the Scottish context, the Scottish Parliament at Holyrood sets the terms of reference and overarching planning permissions for Scottish local authorities. The Scottish Parliament does not yet have control over licensing of oil and gas exploration, but via planning regulations has a de facto veto on UOGE development. Public concern about health and amenity impacts of UOGE were growing. The refusal of coal bed methane (CBM) extraction applications in the central belt of Scotland by the relevant local authorities in 2012–2013 led to industry appeals to the Scottish government, triggering a public enquiry in 2014 [17]. At the enquiry industry and some experts along with commercial health impact consultants argued the economic case for CBM and for its safety. Community groups, councils, environmental groups, some academics, and other commercial health impact assessment consultants argued against CBM development, largely on public health and environmental grounds. 

Before the CBM public enquiry could report, the planning application was “called in” by the Scottish government. This meant that the enquiry would not issue an opinion, allowing ministers in Edinburgh to make a decision on the case. In the UK, Public Health England had already produced a draft report in 2013 and a final report in 2014 indicating UOGE could be done safely if properly regulated and with good industry practice [3]. The Scottish government then set up a panel in 2014 on both unconventional oil and gas extraction, arguing for an “evidence-based” approach to UOGE that would initially be informed by this independent expert panel, whose specialist knowledge largely related to geosciences, engineering, and resource extraction. 

The panel published their report in 2014 claiming “it seems likely that unconventional gas could be developed in the country at a significant scale … none of the particular issues raised by unconventional gas developments would be insurmountable given adequate planning and effective regulation” [18] (p. 18). This allowed the Scottish government some political breathing space but ensured that an understanding of expert opinion and the underlying evidence base informed by those with geo-engineering expertise rather than public health gained currency. The idea that scientists had proclaimed fracking “safe” for Scotland gained some traction and was repeated at every opportunity by industry. However, the terms of reference of that expert panel were to review the environmental and regulatory issues arising from UOGE development, a rather narrower brief than pronouncing on the safety or desirability of such development. 

While acknowledging there might have been gaps to address and more evidence was needed on effects of UOGE, nevertheless it concluded Scotland had the regulatory framework to control UOGE impacts. The panel did not have any public health or legal specialist members, contained no independent expert on industry practice and its final conclusions were challenged by communities and some academics [5]. 

### 3.2. The Scottish Moratorium

Criticism of the report produced by the expert panel [18] may have led to greater circumspection by the Scottish government in tackling the issue of UOGE as it recognized the subject aroused controversy across public and scientific domains. Hence the Scottish government in January 2015 announced a moratorium on granting consents for unconventional oil and gas developments in Scotland while further research and consultation was undertaken. The moratorium process included several interrelated strands, notably a full public consultation on unconventional oil and gas extraction, commissioning of a full public health impact assessment, conducting further work into strengthening planning guidance and considering appropriate environmental regulation [19]. 

While the work on various aspects of the UOGE moratorium was underway the Scottish government decided in October 2016 it would change its planning rules to prevent any underground coal gasification developments in the country because there were concerns about environmental impacts, effective regulation and public health: hence it “had no place in the country’s energy mix” [20] at the time.

The UOGE moratorium reports for fracking and CBM were set within a clear structure and timetable to ensure outcomes, public consultation and parliamentary scrutiny and approval. 

The process was carefully thought through and managed. The report findings would not bind decision-makers as we show in our diagram but would inform debate and consultation. 

#### 3.2.1. The Health Impact Assessment

Health Protection Scotland (HPS), a government agency, conducted a health impact assessment (HIA) of unconventional gas extraction including fracking. It is the most substantial enquiry of the six commissioned by the Scottish government, using input from international referees in a transparent manner, and a systematic literature review of peer-reviewed scientific publications. The conduct of the HIA in terms of setting out criteria for inclusion of research in the review, as well as how expert review of this material was organized, would appear to represent best practice. The very transparency of these processes enabled proper scrutiny of this HIA. Some of our detailed critique, particularly of the drift between the evidence base and the summary conclusions of the HIA, are only possible because the review was conducted with such a high degree of transparency. 

HPS also organized several initial stakeholder events scoping public health issues related to UOGE as part of the wider HIA. There were three dedicated strands: (1) for community groups; (2) industry; and (3) public sector agencies and regulators who may have responsibilities in relation to UOGE. The organization of these events, and how they were to feed into the finding of the inquiry, was an issue of contention for community groups in particular who were dissatisfied with the conduct and framing of this phase of stakeholder engagement. They raised particular concerns over permissions for community groups to submit their own evidence, and disputes over how representative some invitees were of community concerns relating to UOGE. This understanding is based on correspondence between authors and community groups who participated in the stakeholder event and participant observation and is reflected in the official minute of this meeting [21].

The published HIA found evidence for some fracking risks, identified established hazards including ones for workers and likely risks for air and water pollution along with inconclusive evidence for other risks and many data gaps [22]. The report found inadequate evidence to determine whether shale oil and gas or coal bed methane development would harm public health. Based on the available evidence the report acknowledges the relevance of a cautious approach to fracking. Generally, the weighing of evidence is measured and balanced. However, some of the conclusions drawn appear to be optimistic readings of data and experience. For example, assessments of the ability of industry and regulators to control fracking effects on public health do not stand up to scrutiny [23,24,25,26]. Potential resource and staffing problems for regulators and reliance on industry partnerships are noted. The overall thrust of the report on data gaps regarding epidemiological studies of the industry, worker hazards and problems with regulation nevertheless clearly contradicts the industry view that fracking will be safe and highlights potential impacts on densely populated geographical areas. The HIA could not decide on the effects on public health of fracking noise and seismic activity. The report briefly mentions climate change, economic and housing impacts as these are covered in separate enquiries but fails to explore several important studies on the adverse social impacts of fracking especially in terms of stress and well-being [27].

The HIA report offers several conclusions that are not always supported by the evidence it reviewed. Specifically, it downplays some evidence and data gaps, and at times appears to conflate mitigation measures with the application of cautionary approaches rather than separate them in dealing with nation-wide approval of fracking. The available evidence indicates that mitigation through either regulation or good industry practice cannot be assured. Yet the drafting of the report’s conclusions suggests otherwise. Significant relevant literature from peer-reviewed and independent sources on regulation and industry practice is ignored in the HIA and this is a major weakness.

The terms of reference provided by the Scottish government to HPS for the HIA appear to have been changed by HPS. This effectively leads to a downplaying both of a holistic approach to health (which is a central tenet of the Scottish government’s own policies) but also a serious neglect of the literature on social impact assessments and related research and HIA literature that address questions of well-being and mental health. We are unclear to what extent the Scottish government was consulted and agreed these changes. While cumulative health impacts are touched upon in the report, linked to problems of assessing mixtures and interactions of substances, this important subject is still relatively neglected and plays directly into considerations about data gaps and precautionary policies in assessing UGE. 

The downplaying of environmental and social justice issues appears to be another significant weakness of the report. Such questions cannot simply be bracketed out when addressing the hazards, risks, and public health impacts of UOGE. These issues should form an important element of policy consideration in the fracking debate.

The Scottish HIA provides an evidence base for public health concerns and data gaps that the Scottish government considered sufficient to support a decision not to proceed with fracking. To this evidence base one can add research published after the HIA (after 2016) and current knowledge about sustainable energy alternatives. 

#### 3.2.2. Wider Impact Assessments Conducted by the Scottish Government

Evidence from the other Scottish government reports that inform the policy debate are summarized in Table 1 below. A number of these reports either directly or indirectly touch on public health, sometimes beyond their brief and when done by commercial consultants, some seriously downplay risks. Where this occurs is highlighted and briefly discussed next.

Commercial decommissioning consultants analyzed the regulatory framework and stressed the need for adequate funding to ensure that public health was protected when wells were decommissioned. A related issue identified was the importance of long-term monitoring to ensure that any later well failures would be detected and managed. The climate change enquiry did not address public health in detail but unlike other reports appeared to adopt a less complacent approach to regulation and concluded Scotland’s climate change targets would not be met without tighter controls. The transport enquiry considered noise pollution and discovered even with appropriate policies and mitigation “local communities would nevertheless experience an increase in traffic numbers, potentially for some years” [28] (p. iii). However, it concluded planning and related actions could prevent “significant” impacts. A more recent and comprehensive review on noise paints a rather different picture indicating “oil and gas activities produce noise at levels that may increase the risk of adverse health outcomes, including annoyance, sleep disturbance, and cardiovascular disease” [29] (p. 448). This reinforces the need for the latest research to be considered by policy-makers in a field where the evidence base continues to expand. They must also consider the dangers that have emerged elsewhere in the world where “despite significant health concerns, public health knowledge and growing evidence are often overlooked in decision-making” [30].

Seismic activity can create uncertainty and insecurity in communities in many ways that may directly and indirectly affect mental health and well-being. The British Geological Survey conducted the seismic enquiry and found that significant earthquake risks were remote and appeared sanguine that regulatory measures might mitigate any effects from those risks. However, BGS did decide additional data were needed including improved monitoring to understand induced earthquakes and to introduce successful regulatory mitigation measures [31]. While some critics are skeptical about whether and how injection-induced risks might be mitigated, there are yet unresolved debates about seismic activity at particular levels and their impacts.

The economic scenarios commissioned from KPMG attempted to estimate total economic benefits of small-scale fracking activity in Scotland and employment impacts. They concluded a maximum 1400 direct and indirect jobs created, adding 0.1% to Scottish GDP in the coming decades. Even adopting optimistic output assumptions, the scenarios indicate no major benefits to Scotland in terms of middle and long-term economic activity, and hence the associated public health benefits to be gained from jobs and for local businesses appear exaggerated. KPMG, however, did include a section on health-related costs in their analysis and seemingly misunderstood the current state of scientific knowledge. The report claims most fracking chemicals would only have adverse health impacts at “very high concentrations” when much evidence exists on the contrary [32] (p. 37). The generalized claim that most of these chemicals are safe is problematic and does not give the reader any sense of the number, proportion, or volume of chemicals that the industry uses which may not be safe. 

#### 3.2.3. The Public Consultation Exercise

After the reports were published, some four months were set aside for the public to scrutinize them and submit comments. The results of this public consultation exercise after the publication of the moratorium reports were made available in October 2017 [36]. There were 60,535 valid responses which the Scottish government saw as validation of its participative approach. 52,110 (86%) were campaign responses or petitions and 8425 (14%) took the form of substantive responses. For Scottish respondents providing a substantive response with a postcode, almost two-thirds (4151) lived in one of 13 local authority areas identified as potentially having significant shale oil and gas reserves or coal bed methane. Overall, approximately 99% of the responses were opposed to fracking and less than 1% were in favor.

The Scottish Energy Minister’s decision, informed but not dictated by the consultation responses, was that the government would not support any onshore UOGE in Scotland and would prevent it through refusal of planning permissions. This was because powers over licensing applications are reserved to the UK government [37]. This decision was therefore based on the absence of social license for UOGE and, from the moratorium reports, on epidemiological gaps in the literature, negative impacts on Scotland’s climate change targets, weak economic benefits, and likely adverse impacts for communities in densely populated areas of Scotland already affected by old polluting industries. The minister viewed the decision as one based on a precautionary and evidence-based approach but also informed by public consultation. The decision does not mean there will be a formal “legal” ban as some environmental groups and communities hoped for. Nor is it simply an extension of the “moratorium”. However, the Scottish Parliament could yet decide to introduce a National Planning Agreement that would prevent any UGOE occurring in the country for the next five years. INEOS, the major private company promoting fracking in Scotland, has recently signaled its intention to seek a judicial review of the Scottish governments position on UOGE, so some of the process we have analyzed here may also be scrutinized in court. [38] 

#### 3.2.4. The Scottish Government UOGE Review in a Global Context

Shale gas and other oil and gas extraction by fracking occurs in several US states and in some Canadian provinces. It has been approved in England and is contested or banned in many other countries. In Australia, such fracking has been banned in Victoria, and there is a moratorium on conventional onshore gas exploration until 2020 [39]. France has banned fracking due to an application of the precautionary principle [40]. Before shale gas fracking, CBM and UCG projects are permitted it is usual in most states, or provinces and regions where UOGE is proposed for some sort of environmental and health-related impact assessment relating to planning controls to be conducted. These assessments may vary, and some authorities have also produced policies and guidance relating to shale gas extraction before projects are approved. 

In Australia, Canada, USA, and England, for example, a variety of health impact assessments of fracking and CBM or related policy and health reviews have been conducted either by public health professionals, commercial consultants or academics who may be paid by local authorities, government, civil society bodies or industry. Some have looked at specific potential extraction sites and some have attempted to assess the UOGE industry as a whole. The table below lists several of the most influential or revealing of these assessments and related policy documents. While they cover many but not necessarily all the topics addressed by the Scottish government reports, none do so across the board in the depth of the Scottish exercise. The substantial public consultation built into the official Scottish policy deliberation process is very different from the typical public inputs via focus groups or panel surveys etc., run or commissioned by commercial consultants. 

Some of the reports included in the table above offer a very cursory treatment of certain topics. Nonetheless we give recognition where and when the identified topic is at least touched on. In relation to processes around public engagement we acknowledge there are some issues of nomenclature here. In relation to HIA, effective and meaningful “public engagement at all levels should mean much more than simply minimal “stakeholder” involvement that is built into the health impact assessment methodology and sometimes planning requirements. Nor is it covered by “public pushback”, a term used in the USA relating to resistance to fracking proposals. As some of the outputs included for analysis on Table 2 are not strictly HIAs we have sought to capture public engagement as both a theme and practice in our ranking.

Table 2 identifies and rates the key topics and processes involved in UOGE assessments. Our analysis below offers a qualitative assessment of the merits of each and adds some comparative commentary to give context to our assessment of the Scottish government moratorium reviews [33]. 

The report of the Independent Expert Scientific Panel for the Scottish government in 2014 [18] touched on some of the key topics in Table 2. However, there were significant limitations in its coverage of public health, occupational health, regulation and industry practice and a lack of expertise in some of these areas. The panel was comprised of leading engineers, geo-scientists, chemists, and environmental scientists as well as former environmental regulators with considerable experience. This meant that issues related to extraction and production of shale gas were well covered. The panel was not tasked with carrying out public engagement work but significantly noted the importance of such efforts, recognizing “genuine public engagement on unconventional gas needs to include a consideration of social, political and ethical aspects of developments, both within the community and as a nation” [18] (p. 63). The report does not address public health nor examine the related questions of effective enforcement of regulations. It does not adequately cover the issue of resources and staffing needed by regulatory agencies to effectively inspect and oversee the UOGE industry. 

Finkel’s report [2] covered most of the topics identified in Table 2. The background of each chapter author/s was disclosed, and the book included contributions from industry and environmental groups. The book provided one of the most detailed assessments then available of fracking, climate change and public health linked to economic benefits and costs. It does not focus on “vulnerable populations” as such but contributors do cover a range of social determinants of health, economics and employment and endocrine disruptors that ensure the subject is addressed. The publication is unique in that it also has a chapter on impacts on animals in agriculture and the wider environment. It did not, however, pull together all the topics discussed to reach a conclusion about whether fracking should be approved. Nor was any public consultation exercise conducted as this was not relevant at the time to the book’s purpose.

The UK Task Force on Shale [41] was funded by the fracking industry. It produced a series of reports on topics relating to fracking and then an overview document pulling together these disparate elements. A number of the specific reports drew on a range of expertise relevant to assessing the fracking industry. However, the analyses especially of industry practice and in places on regulation were superficial and the coverage of occupational health was deficient. The technical review supporting the final Task Force report does review studies that flag vulnerable populations, social determinants of health, and notes the absence of peer review on some papers. All these qualifications appear to vanish in the final report itself. The Task Force calls for a simplification of the regulatory structure and consolidation of agencies responsible for fracking with the creation of a bespoke regulator. This casts some doubt on whether the current regulatory system in the UK can deal effectively with the considerable health, safety and environmental challenges posed by fracking. 

Werner et al. [42] examined environmental health issues and found direct health outcome evidence on fracking and coal bed methane lacking especially regarding long-term studies. Hence possible health impacts could not be ruled out. The paper specifically addressed societal impacts and reviewed a wide range of papers on government and regulation in peer reviewed and grey publications, recognizing the significance of both subjects to fracking policy. In terms of process, there are considerable strengths in this work, particularly when dealing with uncertainty in scientific findings, and how this is translated in policy processes. 

The US Institute of Medicine [43] published a HIA on shale gas extraction based on a workshop they convened with industry, academics, and government agencies. It did not involve community or worker groups or non-governmental organizations. The publication that arose from that workshop provided information on geographic footprints and one of the most detailed explorations of occupational health and community impacts available at that time. In addition to the necessary sections on air and water pollution, the report also covered sustainable energy and future research. The process used to generate the analyses offered in the report, primarily dominated by scientific and governmental experts in their field has much to commend it. It was not part of the brief to carry out public or community consultation but given the remit and participants it appears it might have been possible to involve health and safety advisors from trade unions directly in the workshop. 

Ben Cave Associates [44] published a HIA that focused on UOGE planning applications in Lancashire county council in the UK. While the focus of this work is at the smallest scale of the studies we have included in Table 2 it merits inclusion as an exemplar of a relatively detailed, careful, and locally sensitive health impact assessment report prepared on fracking. It is generalizable in many respects. The authors were commercial consultants contracted by an English local authority to carry out an assessment determined by planning laws and guidelines that required decisions about fracking mitigation and not whether a fracking proposal should or should not be approved. With relatively limited resources, the process used in preparing this report and handling the evidence available has much validity. The assessment involved 2 community engagement workshops attended by 110 people—for a small scheme, this exceeded the engagement achieved by the Scottish government HIA stakeholder workshop and in the Maryland State report. Also, the Cave report covers several of the key areas either mentioned briefly or missed by better funded outputs. It is especially strong on recommendations for meaningful community input which links with the need for public engagement and trust, and input from the local authority Director of Public Health which links with climate change, environmental justice, and social determinants of health.

The University of Maryland [45] assessment of potential public health impacts associated with UOGE in the Marcellus Shale in Western Maryland is one of two substantial US reports looking at proposal for fracking in part of a state. The authors were all academics and the report was externally peer reviewed. It was prepared for Maryland State Environment and Health Departments. The authors engaged in a scoping exercise with communities which involved two quite small public stakeholder engagement meetings, underpinned by the publication of a draft scoping report as well as contacting industry and local business bodies. The scoping activity is similar to that conducted by the Scottish government exercise and is relatively limited. It also lacks the large public consultation stage that occurred in Scotland.

The report pays particular attention to vulnerable populations, social as well as physical determinants of health, and worker health and safety issues along with discussions of the US and state regulatory landscape. The process used is, within the relevant setting, rigorous and quite comprehensive. 

The HIA produced by Public Health England (PHE) on UOGE in the UK [3] was conducted partly because various national and local agencies requested advice on the matter. The 2013 draft version was produced following statements by UK government ministers supporting fracking with appropriate caveats about the industry being properly regulated and following good practice. PHE is a government executive agency of the UK Department of Health. Although it states it has operational autonomy in 2015 it was criticized in the BMJ as being “nominally independent, (but) appeared to be serving the policy agenda of a government promoting the potential of fracking…to provide the UK with greater energy security, growth and jobs” [51]. The PHE review excluded consideration of climate change and greenhouse gas emissions, sustainable use of water resources, nuisance issues, traffic (apart from vehicle exhaust emissions), occupational health, visual impact and the socioeconomic benefits and impacts of shale gas extraction. A BMJ commentary on the report noted that “a focus on mostly hypothetical regulatory and engineering solutions may mistake best practices for actual practices and supplants the empirical with the theoretical” [23]. The report has some but limited relevance in informing a comprehensive policy process to assess fracking in 2018. Despite several key weaknesses including neglect of mental health, no consideration of cumulative exposures and little analysis of industry practice under different regulatory regimes [5] (pp. 26–29) the PHE report has been politically significant and has been cited repeatedly by politicians and industry to claim that fracking can and will be conducted safely in the UK.

The public health review of high volume UOGE conducted by the New York State Department of Health [46] is a relatively detailed and lengthy assessment of the subject. It was conducted drawing on a wide range of staff with extensive expertise in the fields of public health, science, and medicine. The resources devoted to this report exceeded those made available for example in the Scottish government public health report of 2017 although in some respects the scope was more limited. There is relatively little detail on industry practice. Like other reports, it highlights gaps in knowledge about impacts and adopts a cautionary approach. Such an approach is not obvious in several other reports we reviewed. Most other studies reviewed here do not display this range and level of detail. Its strength lies in the interrogation of research methods and assumptions built in to research designs. Skepticism and rigor are hallmarks of the evaluation of results and the methods that produced those results (including interpretation of secondary data analysis and whether data warrant some of the respective authors’ conclusions). The report is very relevant to the policy-making process and contains transferable examples of good practice.

The Bloomfield AEA plc report for the European Commission [47] is an extensive, careful, and cautious study done by commercial consultants for the European Commission. It drew heavily on North America information where fracking was already underway but set its findings in the context of European regulatory and legislative environment. Serious potential risks with fracking are identified and discussed, which stands in contrast to the apparent exclusion of this type of consideration by the UK government. The report emphasized and drew strongly on peer reviewed scientific reports, though there is little or often no peer reviewed literature to support judgements about regulatory effectiveness and industry good practice in fracking. These topics may have been considered marginal to the main focus of the report. In this context, the publication has limited value to the “process” of producing comprehensive assessments of the industry. There is some recognition of fracking impacts on local populations through “operators working cooperatively with regulatory agencies and other stakeholders to promote best practices and improve communication with local communities” [47] (p. 138). However, this does not relate to community participation in fracking approvals. The report therefore has both strengths and weaknesses in informing the policy process.

The recommendations of the Office of the Chief Medical Officer of Health in New Brunswick [48] is a relatively concise report reviewing the scientific literature on hazards and health impacts assessments of shale gas production. It was prepared by the Chief Medical Officer to identify both the state of knowledge and gaps in knowledge of shale gas with relevant recommendations. It is, therefore, similar in some respects to the APHA policy statement [49] and in terms of “process” does not fall neatly into a category where a template could be devised. However, several principles it draws on and applies in its recommendations on health protection would be central to an effective assessment process. These are present in other reviewed reports but not necessarily highlighted in the same way or emphasized so strongly. They include discussion of health equity, transparency and community participation, vulnerable populations especially children linked to protecting future generations and, in a Canadian context, First Nations peoples. In Scotland such groups would include those in former mining communities and heavily populated polluted de-industrialized manufacturing areas that are not often perceived as containing vulnerable populations at all by industry. The emphasis in the report on ethical consideration, values, and principles to “guide all actions to improve, promote and protect health” [48] (p. 4) is surprisingly missing in several of the other documents we reviewed. These should be guiding principles in the process adopted to assess fracking and its impacts. 

The North American Public Health Association (APHA) [49] policy statement on Environmental and Occupational Health Impacts of High-Volume Hydraulic Fracturing of Unconventional Gas Reserves ranges over the whole fracking process including site preparation, drilling, and casing, well completion, production, transportation, storage and disposal of wastewater and chemicals, and site remediation. It additionally includes occupational health and safety, climate change and economic aspects. Other reports have sometimes had a much narrower focus on well construction and immediate production. It identified a wide range of short-, medium-, and long-term risks to a range of populations. While the statement lacks some of the depth of other reports listed in Table 2, the process used and the comprehensive nature of the topics covered may be recommended. The APHA highlight the importance of cumulative health impacts and good quality base line health data to inform policy and flag the necessity of involving public health professionals in regulatory assessments and decision-making processes.

Witter et al. [50] prepared a substantial HIA for Battlement Mesa, Garfield County, a local shale gas proposal within Colorado. It was one of the earliest documents on this subject. The HIA runs to over 110 pages with appendices and was partly supported by Garfield County and by two charities. Unlike several more limited UK reports at the time on similar subjects, shaped by the narrow planning requirements and often commissioned by industry, the process used in this HIA ensured a comprehensive assessment of the proposed industry development. The scoping exercise conducted by the team included meetings with citizens, the industry, regulators, and the state health department. Seven stakeholder meetings were held over a six-month period. The assessment used its own 2008 Community Health Risk Analysis of the Oil and Gas Industry report relating to Garfield County. This had already collected baseline air monitoring data for the area that it noted even then increased risks of negative health effects on citizens. The process used by the Witter team, drawing on these detailed baseline pollution and health data, is a model for future assessments that was often never followed. It contained considerations of vulnerable populations, social determinants of health, community wellness and social cohesion—very often neglected elsewhere in other reports—and economic impacts. The process finally threw up recommendations on tighter pollution prevention and better monitoring, public safety, boomtown problems, tougher regulation and guarantees of transparency. The report provides an excellent template to inform the policy process.

Our assessment of the relative strengths of these reports leads us to conclude that the process underpinning the Scottish government public health review has much to recommend it. For example, time spent on the review, the range of evidence included, the resources devoted to the individual strands of evidence gathering, as well as the very significant commitment to public consultation, together clearly show the Scottish approach is more comprehensive and integrated and de facto “better” than other approaches to date. We base this judgement on our reading of the available policy reports under the criteria we have outlined in Table 2. This framework highlights scope and coverage but does not discriminate in terms of depth or efficacy, beyond the simple ranking we have offered. While we think that the criteria we have foregrounded in Table 2 are helpful to policy makers and stakeholders interested in UOGE, judgements about how well individual reports and assessments meet these criteria are not easily scored or translated into agreed metrics. As such we have devoted some space to adding some description of the reports selected to give readers a clearer sense of how we developed our analysis and warranted our claims about the deliberative processes that have informed Scottish policy. 

## 4. Discussion

The Scottish government approach to UOGE evolved over several years in response to political pressures from a range of industries, community groups and environmental NGOs. Initially this allowed a difficult policy choice to be shelved, meaning that a decision that was sure to alienate one side or another of the argument was postponed. However, the moratorium that finally emerged did provide a substantial and integrated set of findings to inform ministers, all interested parties and the wider public. 

While there are certainly weaknesses in the Scottish government review, for instance in relation to aspects of the public health impact assessment and initial engagement by HPS with community groups, the absence of dedicated environmental impact assessments and consideration of cumulative exposures, the template appears to be largely replicable and lessons can be learned from this approach in other jurisdictions grappling with policy and decision making on UOGE. Future research on this subject in the Scottish context may reveal in more detail how government responded during the moratorium process to a range of stakeholders with very different assessments of UOGE. Of particular interest will be the public health concerns articulated by communities and environmental groups as well as politicians at a national and local level. So too will be the arguments used by UOGE industries who expressed confidence in their own production standards, the economic prospects for UOG development, the regulatory system and the mitigation of risk posed by industry to public and worker health and safety. The mechanics of setting up the various groups to prepare the reports and their membership has also not yet been fully revealed. With such information, it may become clearer how exactly the Scottish government weighted gaps about UOGE epidemiology, limited economic benefits, climate change and the need for a social license in their decision-making. 

Our purpose in this paper is not to second-guess how or why evidence was understood and integrated into political decision making by politicians, officials, and regulators. We are of course aware of the very real limits to evidence-based policy making and how data maybe be variously interpreted by different actors at any moment in the political cycle. What we have tried to do in this paper is to draw out the process and areas of good practice that may help ensure decision making around the contentious development of UOGE is *more likely* to be informed by the available evidence, and *more likely* to command the respect or compliance of all stakeholders. We do not make any absolute claims for the rationality of the policy process, though we do clearly insist that independent scientific evidence should be central to public deliberation on issues at the interstices of science, technology, and well-being. Our emphasis on the independence of scientific advice means that we need to treat industry and regulator self-assessments with some care and understand that economic and political interests cannot simply be evacuated from the analysis. It appears to us that the “deficit” model of public understanding of science is also a caricature, and that community groups and the wider public are perfectly capable of engaging with technical detail and scientific uncertainty. It is, therefore, incumbent on those organizing public consultation and dialogue surrounding issues such as UOGE to make every effort to ensure that scientific knowledge, and the limits to that knowledge, are communicated clearly and effectively. 

The 2014 HIA undertaken in New York State, while less detailed than that of the Scottish government review in some respects, nevertheless found many data gaps relating to fracking. They determined gas extraction had already caused environmental impacts potentially damaging to public health based on scientific evidence available at that time and so decided fracking should therefore not proceed in the state [46]. The state of Maryland, which has significant shale reserves, has also recently banned fracking. Onshore UOGE has also been banned in Ireland, France, Germany, and Bulgaria [2,4,40]. 

The publications reviewed all contain examples at various levels of detail of processes that explored potential air, water and soil pollution effects from fracking and the means available to mitigate those effects. Some, however, provided better examples than others of a process that brought together key public health principles to inform and produce comprehensive assessments. The Colorado [50] and Cave [44] publications did so for “small-scale” fracking projects. The APHA [49], New York State [46] and New Brunswick [48] publications did so for larger schemes. The Scottish government initiative [33], while not underpinned by the detail or ethical thrust of some of the other assessments, provides a process that appears to represent the best and most comprehensive national approach yet implemented. 

In terms of process it is clear that the Scottish review considered many of the key policy reports and existing HIAs conducted in other jurisdictions. In terms of original research there was a reliance on the emerging evidence base in places where fracking was underway. Some detailed research had been carried out on potential water pollution by the United States Environmental Protection Agency, but only after fracking had begun and after stakeholder pressure in response to a disputed draft of the report was released. Community groups across Scotland were keen to stress the precautionary lessons to be learned from such US experience. It was noted that states such as Pennsylvania do not appear to have fully assessed health impact issues or regulation, years after permitting UOGE development. Assessments of public health and UOGE undertaken in Colorado [50,52] and Maryland [45] provided more substantive issues to consider in Scotland. The latter state also carried out some extensive stakeholder engagement including over 40 interviews with key stakeholders. The initial HPS stakeholder engagement workshops in Scotland in late 2015 provoked some concerns over the transparency of how community groups and ‘lay stakeholders’ were identified and selected [22]. Alongside community stakeholder engagement, there were also dedicated workshops for industry stakeholders, and also one comprising public sector agencies. Stakeholders were also invited to nominate expert peer reviewers who would assess the existing evidence on health impacts of UOGE. The Scottish HIA relied on four reviewers, three of whom were nominated by stakeholders (one by industry, and two by different community interest groups) [22] (p. 16). The rationale for this approach rests on the recognition that ‘different stakeholders may therefore interpret the same evidence in different ways and draw different conclusions, depending on their original perspective’ [22] (p. 28). The Scottish stakeholder engagement workshops offered the key opportunity for external organizations and interests to influence the HIA. The potentially affected populations identified by these workshops included groups one might typically consider impacted by such developments, namely nearby residents, vulnerable groups and workers. However, the workshops also identified those working for agencies charged with regulating UOGE as an impacted population, and registered concerns about ‘overstretch’ of those professionals carrying out their regulatory duties [22] (p. 38). The Scottish government national consultation exercise run in 2017 attracted 60,535 valid responses of which 52,110 (86%) were campaign responses or petitions; and 8425 (14%) took the form of substantive responses. Of those respondents in Scotland providing a substantive response, and a postcode, nearly two-thirds (4151) lived in one of 13 local authority areas identified as potentially having significant shale oil and gas reserves or coal bed methane [37].

The evidence collected for the Scottish government enquiry suggests there are significant public health risks and costs from UOGE including fracking and coal bed methane with only 0.1% added annually on average to Scottish GDP if fracking went ahead [32]. Employment in this sector is likely to require a mobile workforce who will not necessarily reside in areas where UOGE is conducted, though this workforce will contribute to the local economies where they are based during production. While the economic projections surrounding UOGE development in Scotland are contested, there is a recognition that the benefits of UOGE are unlikely to directly accrue to local populations, even allowing for the community benefit packages developers have proposed for impacted communities to help secure planning consent. An earlier report commissioned by the Scottish government also raised many questions about the health and other impacts of underground coal gasification and so the government decided not to proceed with that technology [53]. Hence the case for fracking, coal bed methane and underground coal gasification looks difficult to sustain. Between 2015 and 2017, research and reviews have emerged that cast even more doubt on UOGE regarding noise, mental health, well-being, worker safety, air and water pollution, birth outcomes, hospitalization rates, childhood cancers, asthma, neurological effects, and perinatal outcomes [54,55,56,57,58,59,60,61,62,63,64]. 

Important questions about the nature, scope and commissioning of the Scottish reports that inform the policy making process and their relative weighting and integration into the wider public health assessment deserve some consideration. Resources and timescales may shape outcomes as may wider political drivers. The Scottish enquiries, taken together (as they were intended to be), still look substantial. Their terms of reference are critical, and the neglect of dedicated environmental impact assessments appears an obvious omission. There is some evidence that terms of reference were changed in the HIAUOGS in ways that privileged the discussion of physical hazards and risks over psycho-social ones by separating off direct and indirect health effects. 

There is little detailed consideration of cumulative health impacts or complex interactions that may be involved in fracking [65,66]. The exposome concept [67] developed in 2005, to look at total environmental exposures during a life time would also appear to be highly relevant to impact assessments in heavily industrialized areas where fracking is underway or proposed. It is unclear if such an approach was ever considered for the Scottish review. 

Concerns persist over how HPS handled stakeholder engagement in their scoping of the HIA. These must be balanced against the widespread consensus achieved and legitimacy accorded to the subsequent national consultation process overseen by the Scottish government. Arguably it is better to have widespread public consultation on detailed proposals and evidence rather than extensive stakeholder input at the beginning which can be ignored at later stages. However, given that community relations have been put at the center of governance and regulation of UOGE in Scotland [5,22,37], and indeed the rest of the UK [5,24,41], it is important that these processes are managed equitably and openly.

With all its blemishes, the Scottish approach in terms of process, if not all its outcomes, may prove to be a valuable template to use elsewhere in the world. The Scottish process involved a greater breadth of disciplines than the process conducted in England. The Royal Society and Royal Academy of Engineering review of hydraulic fracturing in 2012 [68] recognized the uncertainty that would accompany large-scale production activity and identified knowledge gaps as well as the need for baseline health data. Public Health is largely ignored in this RA/RSE report and the expertise of the panel members did not extend to medicine, public health or indeed anyone with independent expertise in scrutinizing industry practice. Despite these omissions , and the by now somewhat dated evidence base, the RA/RSE report has been, and continues to be, repeatedly relied on in UK policy discourse to affirm the safety of UOGE. Given ongoing protests, planning objections and legal appeals in parts of England where fracking is proposed it is clear that effective public engagement is lacking [69] and there is no “social license” to frack [4,26]. However, while the Scottish process used to weigh the evidence has much to commend it, those responsible for the careful drafting of the final public health report left some leeway for politicians to make their own call on this sensitive issue. In this respect, the Scottish report differs, from the New York State report which arrived at a clear conclusion against UOGE. Whether this is due to differences in perceived mandate and scope or differing perceptions as to the weight of the evidence is not clear. If national or regional policies are being made on UOGE, it is legitimate to ask those assessing the available evidence to answer directly whether UOGE can be conducted in ways that will not endanger public health? If the answer to that question, based on the latest research evidence, is “no”, then debates about mitigation, community engagement and consultation are moot. 

## 5. Conclusions

While not a perfect mechanism nor a completely exhaustive set of findings—which cannot anyway be generated at the present time with the state of current knowledge—the Scottish governments approach to assessing the impact of UOGE development was found to comprehensively address all ten attributes considered in this study. No other studies examined here address all ten aspects of UOGE development, despite every effort to locate the most relevant and complete studies which include assessments undertaken within the USA, Canada and Germany. It is the first truly national assessment of the public health and related implications of UOGE

We would argue that the approach used in Scotland should be largely transferable globally, despite differences in energy needs, energy policies, geology and water resources, demography (especially population density), planning and regulatory laws. The very latest international research has raised even more questions about both CBM and fracking impacts on air, water and soil and noise and transport pollution [4]. As research and the existing knowledge base on UOGE impacts continues to develop, informing policy and decision making with the latest evidence is clearly a significant issue. We believe that lessons can be drawn from the Scottish case on how to meet these challenges. 

## Figures and Tables

**Table 1:** Scottish Government Reports commissioned during the UGE moratorium 2015–2016 [33].

Subject of Reports—All Published in the Winter of 2016	Author	Key Findings
A Health Impact Assessment of Unconventional Oil and Gas in Scotland (HIAUOGS) [22]	Health Protection Scotland—NHS Scotland	Generic assessment. “Sufficient evidence” on a number of likely air and water environmental hazards and silica hazards to workers. Other evidence inadequate to decide if process poses a public health risk.
Climate change impacts [34]	Committee on Climate Change—independent body established under the Climate Change Act (2008) to advise the UK Government	Only compatible with Scotland’s climate change targets if (a) emissions limited through tight regulation; (b) Scottish UOGE production displaces imports (c) emissions from production of UOGE offset by reductions in other emissions in Scottish economy.
Decommissioning, site restoration and aftercare—obligations and treatment of financial liabilities [35]	AECOM—commercial consultant (provides design engineering services to oil and gas industry)	Assumes best practice and appropriate regulation and monitoring will ensure decommissioning succeeds. Notes a residual risk that a small proportion of wells may fail.
Understanding and mitigating community level impacts from transportation [28]	Ricardo Energy and Environment—commercial consultant	Assumes appropriate strategic policies are put in place, and appropriate mitigation is carried out, local communities would nevertheless experience an increase in traffic numbers, potentially for some years.
Understanding and monitoring induced seismic activity [31]	British Geological Survey—advises UK government on all aspects of geoscience, also provides geological advice to industry, academia, and the public	USA and Canada evidence suggests probability of induced earthquakes felt is small, although some examples exist of earthquakes large enough to be felt.
Economic impacts and scenario development [32]	KPMG	1400 jobs created at peak in the Scottish economy bringing in, on average per year, 0.1% of Scottish GDP. Impacts on local house prices, road use, agriculture, visual amenity, environmental costs, and health costs.

**Table 2:** UOGE “Health Impact Assessments”/Policy: topic coverage and *process overview.*

	Occup. Health	Climate Change	Regulation	Industry Practice	Economics and Employment	Vulnerable Populations	Social Determinants of Health	Peer Review	Decl of Interest	Public Engagement
Scottish Government (2016) [33]	√	√	√	√	√	√	√	√	√	√
Scottish Government (2014) [18]	.	√	√	.	.	x	.	x	√	.
Finkel (2015) [2]	√	√	√	√	√	.	√	n/a	n/a	√
Task Force on Shale (2015) [41]	.	√	√	.	√	x	x	x	x	√
Werner (2015) [42]	√	.	√	.	.	√	√	√	√	n/a
US Institute of Medicine (2014) [43]	√	√	√	√	√	√	√	√	n/a	x
Ben Cave Associates (2014) [44]	√	√	√	√	√	√	√	n/a	√	√
Maryland Univ (2014) [45]	√	√	√	√	√	√	√	√	√	x
PHE (2014) [3]	.		√	.	.	x	x	x	n/a	n/a
New York State (2014) [46]	√	√	√	√	.	x	√	√	n/a	.
AEA (2012) [47]	√	√	.	√	.	x	x	x	n/a	n/a
New Brunswick (2012) [48]	√		√	√	√	√	√		n/a	.
APHA (2012) [49]	√	√	√	√	√	√	.	√	n/a	x
Colorado Univ (2010) [50]	√	x	.	√	.	√	√	√	√	√

√—discussed in some detail; n/a—not applicable; .—mentioned briefly/superficial treatment; x—not covered directly.

## Abbreviations

CBM	Coal Bed Methane
GDP	Gross Domestic Product
HIA	Health Impact Assessment
HIAUOGS	Health Impact Assessment of Unconventional Oil and Gas in Scotland
HPS	Health Protection Scotland
UCG	Underground Coal Gasification
UOGE	Unconventional Oil and Gas Extraction
UOG	Unconventional Oil and Gas
